# Evolutionary innovation through fusion of sequences from across the tree of life

**DOI:** 10.1073/pnas.2602557123

**Published:** 2026-07-13

**Authors:** Rishabh R. Kapoor, Evelyn E. Schwager, Supanat Phuangphong, Emily L. Rivard, Chandrashekar Kuyyamudi, Suhrid Ghosh, Isobel Ronai, Cassandra G. Extavour

**Affiliations:** ^a^ Systems, Synthetic, and Quantitative Biology Program, Harvard Medical School, Harvard University, Boston, MA 02115; ^b^HHMI, Chevy Chase, MD 20815; ^c^ Department of Organismic and Evolutionary Biology, Harvard University, Cambridge, MA 02138; ^d^ Department of Molecular and Cellular Biology, Harvard University, Cambridge, MA 02138

**Keywords:** molecular evolution, horizontal gene transfer, gene fusion, arthropods

## Abstract

Novel genes arise through multiple mechanisms, including gene duplication, gene fusion, and horizontal gene transfer (HGT). While HGT has increasingly been documented in animals, the posttransfer evolutionary fate of horizontally acquired genes is less well understood. We hypothesized that fusion with endogenous sequences in animal genomes might generate what we call “HGT-chimeras”: genes with regions of nonmetazoan and metazoan descent in the same open reading frame. To test this hypothesis, we developed a molecular phylogenetics pipeline that enables the identification of HGT-chimeras. We applied our pipeline to 319 high-quality annotated arthropod genomes and uncovered a high-confidence set of 274 HGT-chimeras corresponding to 104 independent origination events across diverse arthropods. HGT-chimeras contain intervals acquired from across the tree of life, and many likely originated via a gene duplication-based mechanism. To assess whether HGT-chimeras might be functionally important, we performed RT-PCR and Sanger sequencing of tissues from 20 arthropod species predicted to harbor HGT-chimeras in their genome. We found evidence for the expression of contiguous chimeric messenger RNA transcripts (mRNAs) for 36 of 41 tested HGT-chimeras across 18 of 20 different tested species. We also found evidence that HGT-chimeras evolve under purifying selection and have acquired potentially functional domain architectures, consistent with the hypothesis that these genes are in active use and may participate in diverse biological processes. These results illuminate an underappreciated combinatorial mechanism underlying the origin of novel genes across the largest animal phylum, and suggest that interdomain sequence fusion can play important roles in animal biology and evolution.

## Significance

Evolution forges novelty through the repurposing of available parts. Can recently acquired parts, previously foreign to an organism, be similarly repurposed? Applying a rigorous methodology to 319 genomes from arthropods, the largest phylum of animals, we uncover 104 novel genes that arose from the fusion of animal genes with fragments acquired via horizontal gene transfer from bacteria, viruses, fungi, and plants. RNA sequencing and RT-PCR across multiple species show that many of these novel genes are expressed as mRNAs. Many show signatures of evolutionary conservation and coherent domain architectures, suggesting that these chimeric genes may play roles in diverse biological processes. These results reveal an understudied path to evolutionary innovation via “bricolage” of genes from across the tree of life.

Novel genes contribute to adaptation and essential biological functions across the tree of life (1, 2), and have emerged through mechanisms such as duplication (1–3), fission (1, 2), fusion (4–8), and de novo birth (9, 10). Among these mechanisms, horizontal gene transfer (HGT) allows organisms to acquire new genes in a non-Mendelian fashion from other species (3, 11–15) and is widely appreciated as a fundamental force of evolution in prokaryotes (12). Beyond an initial burst of HGT during early stages of eukaryotic symbiogenesis, HGT was long assumed to be vanishingly rare and therefore unimportant in eukaryotic evolution (3, 11, 16). Arthropods, the phylum of animals that constitute at least 80% of all described animal species (17), are emerging as an exception to this expectation. Horizontally acquired genes from bacteria (13–15, 18–23), viruses (14, 24–26), fungi (14, 27, 28), and even plants (14, 29) have been identified in diverse arthropods. In many cases, these genes contribute adaptive functions ranging from ecological defense (23, 29) to metabolism (28, 30). A recent systematic screen for HGT across 218 insect genomes uncovered 741 independent HGT events, placing the rate of HGT on par with that of other rare novel gene formation events within this phylum (14).

Whether horizontally acquired genes retain similar gene structures and functions following transfer, or, like other kinds of novel genes (7, 31–34), rapidly diverge from their original forms, has received less attention than detection of the transfer phenomenon. Here we focus on gene fusion as a possible mechanism of posttransfer divergence. Fusion of endogenous gene sequences is a well-documented source of novelty in protein structure and function (4–8), but has less often been systematically examined across hundreds of eukaryotic genomes (although see refs. 35–38). We hypothesized that, as a result of the fusion of horizontally acquired sequences with preexisting host genome sequences, animal genomes would contain “HGT-chimeras”: genes with at least one region horizontally transferred from a nonmetazoan source in the same open reading frame as a region of ancient metazoan ancestry. To our knowledge, no large-scale systematic screen for HGT-chimeras has been conducted in multicellular eukaryotes, but a few examples have been identified in gene family-specific studies across diverse taxa (21, 39–42). For instance, we previously attributed the origin of *oskar*, a gene required for essential developmental processes in diverse insect species (43–46), to the formation of a HGT-chimera between a metazoan LOTUS domain (47) and a bacterial GDSL hydrolase domain (48, 49) in the last common ancestor of extant insects (50). In another example, a screen of 20 microbial genomes identified 37 putative interkingdom HGT-chimeras in 11 unicellular species, but did not examine multicellular eukaryotes (51). Here, we present the results of a systematic bioinformatic screen for HGT-chimeras across 319 arthropod genomes. We find evidence that HGT-chimeric genes are not only widespread across the tree of arthropods, but also display transcriptional and molecular evolutionary characteristics that suggest they play active roles in organismal biology and evolution.

## Results and Discussion

### Development of an HGT-Chimera Detection Pipeline.

**Fig. 1.**
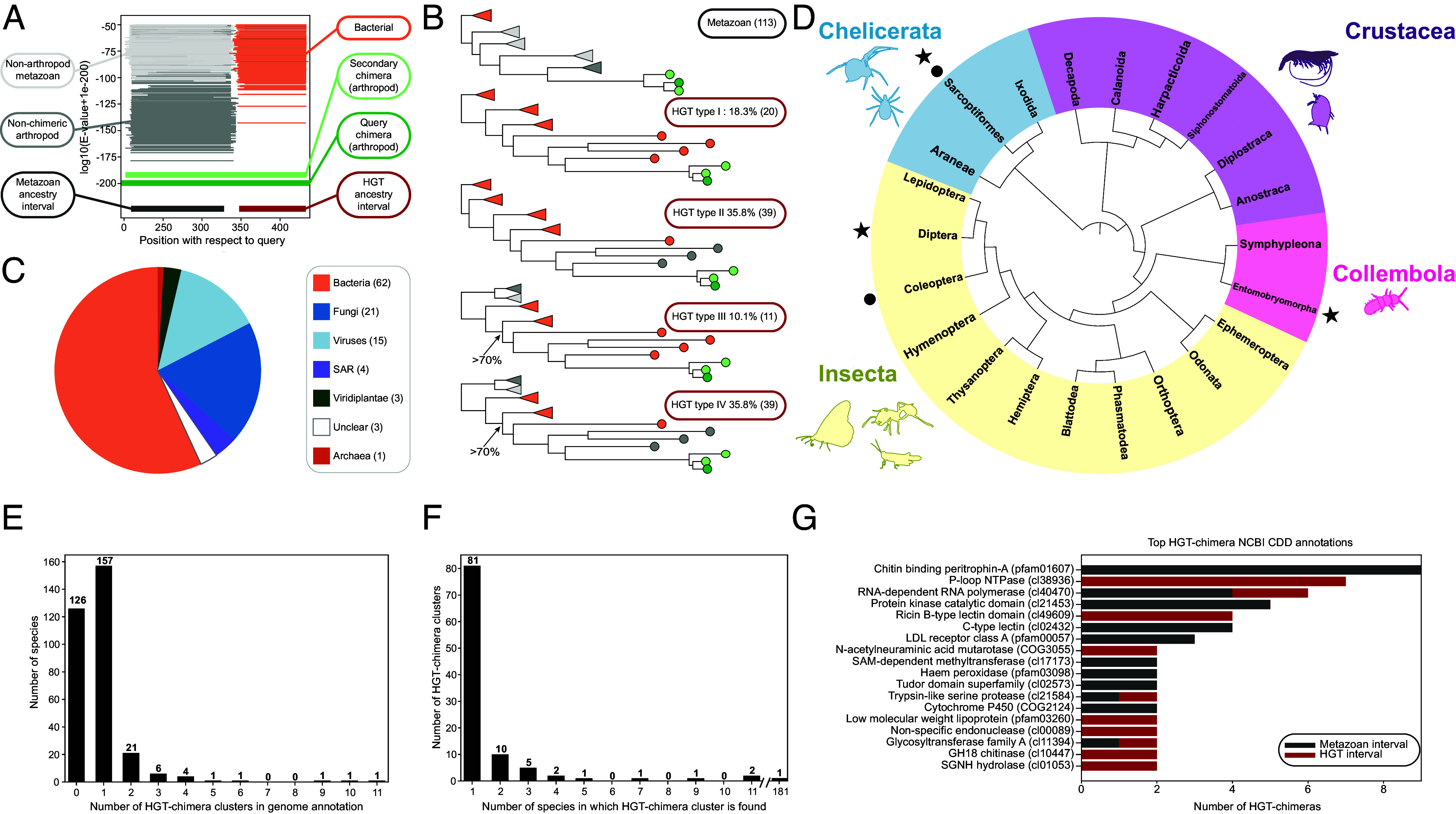
HGT-chimera detection across Arthropoda. (*A*) Illustration of the taxonomic distribution of BLASTp hits (colored thin horizontal lines) across a hypothetical HGT-chimera query sequence (dark green). Dark gray and red bars across the bottom indicate inferred metazoan and HGT ancestry, respectively. In addition to the original query sequence (dark green), secondary chimeric sequences of the same domain architecture (light green) are retrieved from other arthropods. Plots for all HGT-chimeras are available in *SI Appendix*, *SI File 1*. (*B*) Hypothetical examples of maximum likelihood tree topologies for HGT-chimera intervals assigned to metazoan and HGT ancestry, with HGT-supporting topologies assigned to four subcategories (*SI Appendix*, *SI Methods*). Proportion of intervals assigned to each type in the final set of representative HGT-chimeras are indicated, with numbers in parentheses. (*C*) Plausible HGT donor taxa at the domain/superkingdom level inferred from the taxonomic distribution of sister and cousin clades on maximum likelihood phylogenies for 109 HGT intervals from 104 HGT-chimera clusters (*SI Appendix*, *SI Methods*). SAR refers to the eukaryotic supergroup consisting of stramenopiles, alveolates, and rhizarians. (*D*) Arthropod orders with species containing at least one HGT-chimera. Colored ranges indicate taxonomic class (Insecta, Collembola) or subphylum (Chelicerata, Crustacea). Symbols outside the phylogram indicate the presence of the sparsely distributed HGT-chimera clusters 18 (circles) and 3 (stars). (*E*) Distribution of the number of HGT-chimera clusters detected per RefSeq genome. (*F*) The number of species possessing each of 104 HGT-chimera clusters. (*D* and *F*) contain data from both the primary search set (RefSeq genomes) and GenBank genomes, while (*E*) contains data only from the former. (*G*) Counts of the top NCBI Conserved Domain Database (CDD) annotations assigned to representative HGT-chimeras. Bars are colored by whether the annotation occurs in an HGT or metazoan interval. Names have been abbreviated, with CDD accessions provided in parentheses.

**Fig. 2.**
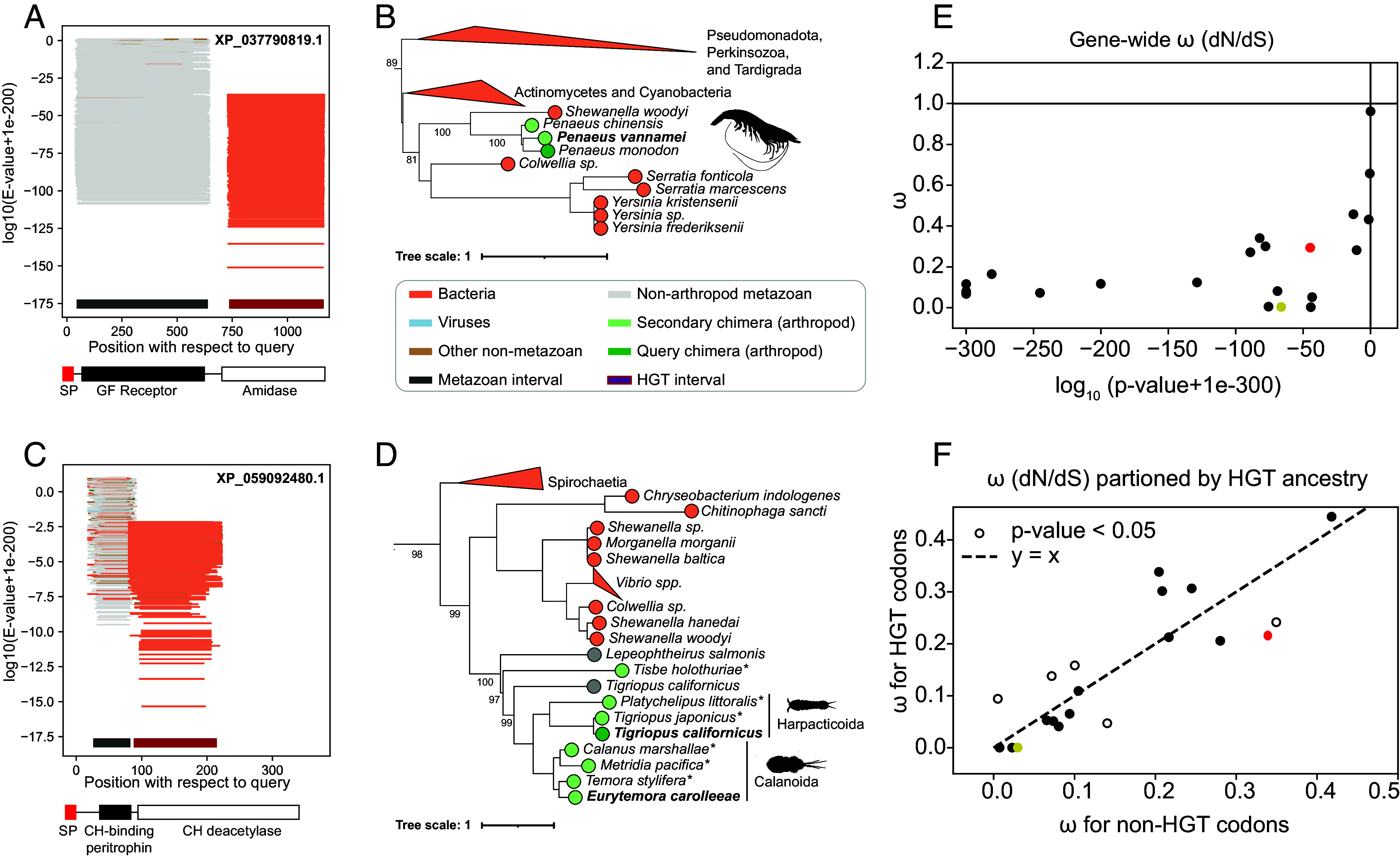
Crustacean chimeras of varying age have evolved under purifying selection. (*A*) BLASTp plot as in Fig. 1*A*, but excluding arthropod hits, for the query chimera XP_037790819.1 from the shrimp *Penaeus monodon* (cluster 12). Metazoan ancestry is inferred for the interval (45 to 640) and HGT ancestry for the interval (736 to 1,165). Domain annotations are shown across the bottom of the plot, along with an N-terminal signal sequence (cleavage site between positions 25 and 26). See *SI Appendix*, Fig. S11 for detailed annotation. (*B*) Maximum likelihood tree for the HGT interval (736 to 1,165) from XP_037790819.1, including intervals from secondary chimeras found in the two other congeneric shrimp *Penaeus vannamei* (XP_069990332.1) and **Penaeus* chinensis* (XP_047489075.1). (*C*) BLASTp plot excluding arthropod hits for the query chimera XP_059092480.1 (cluster 14) from the copepod *Tigriopus californicus*. Metazoan ancestry is inferred for the interval (26 to 83) and HGT ancestry for the interval (88 to 215). Domain annotations are shown across the bottom of the plot, with an N-terminal signal peptide cleaved between positions 29 and 30. See *SI Appendix*, Fig. S12 for detailed annotation. (*D*) Maximum likelihood tree for the HGT interval (88 to 215), including secondary chimera sequences from the genome of the copepod *Eurytemora carolleeae* (XP_023343432.1) and additional sequences obtained from transcriptome shotgun assembly data from six additional copepod species (indicated with an asterisk). The phylogenetic split among HGT-chimeras from the copepod orders Harpacticoida and Calanoida is indicated with vertical lines. In *B* and *D*, collapsed multisequence clades are represented as triangles. Triangle color represents the taxonomic origin of the majority of contained sequences, and the lengths of the two edges connecting each triangle to its parent node represents the summed branch length to the longest and shortest branch in the clade. Bolded text in *B* and *D* indicate sequences verified by RT-PCR, scale bars indicate one substitution per amino acid site, and numerical values at internal nodes indicate ultrafast bootstrap support values for select nodes relevant for HGT inference. Only a portion of both trees is shown here; the full trees for both metazoan and HGT trees with accessions are available on iTOL (57). (*E*) Gene-wide ω (dN/dS) values fitted for 21 of the 23 HGT-chimera similarity clusters that were found in the genomes of at least two different species (excluding clusters 3 and 18). The x-axis represents the log-transformed Benjamini–Hochberg corrected LRT *P*-values. (*F*) Each of 23 HGT-derived intervals from the same 21 HGT-chimeras as in *E* were permitted to take a different ω value from the rest of the protein using fixed-sites models. Open dots indicate statistically significant deviation of dN/dS values for an HGT-derived interval from the rest of the coding region (Benjamini–Hochberg adjusted LRT *P*-value < 0.05). Three intervals with y-axis values > 0.50 and *P*_adj > 0.05 have been omitted from this plot. Red and yellow circles in *E* and *F* highlight clusters 12 and 14, respectively.

**Fig. 3.**
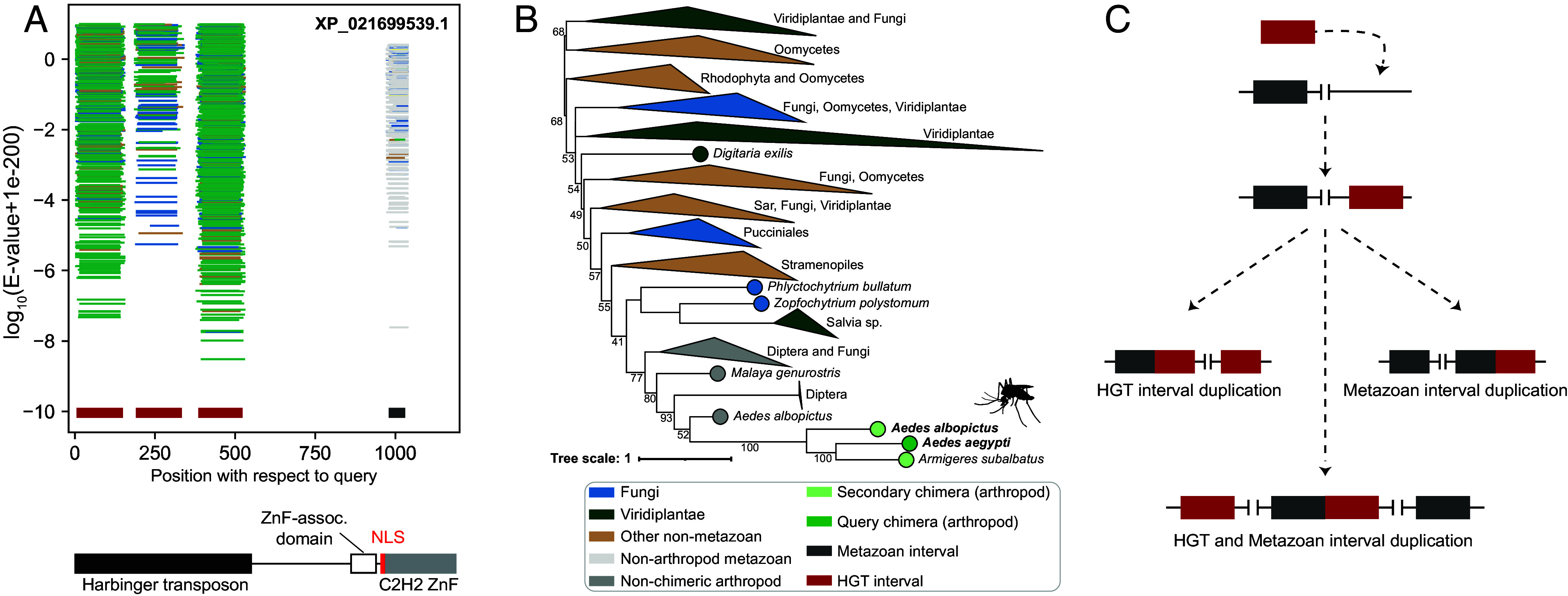
An HGT-chimera from mosquitoes illustrates that nonchimeric HGT can precede HGT-chimera formation. (*A*) BLASTp plot excluding arthropod hits for the query chimera XP_021699539.1 from the mosquito *Aedes aegypti* (cluster 9). Metazoan ancestry is inferred for the interval (980 to 1,031) and HGT ancestry for the intervals (5 to 150), (190 to 334), and (385 to 523). Domain annotations are shown across the bottom of the plot, along with a nuclear localization sequence from residues 975 to 985. See *SI Appendix*, Fig. S13 for detailed annotation. (*B*) Full maximum likelihood tree for the HGT interval (190 to 394), including secondary chimeras from the mosquitoes *Armigeres subalbatus* (XP_062544500.1) and *Aedes albopictus* (XP_029735553.1). Bolded text indicates that the chimera was successfully verified via RT-PCR. Collapsed clades are represented as in Fig. 2. (*C*) Schematic representation of a model of HGT-chimera formation in which HGT precedes HGT-chimera formation, which may involve gene duplication. This model posits the existence of nonchimeric relatives to the HGT or metazoan intervals within the same genome or in the genomes of closely related arthropods. Note that the expected outcome of fusion via tandem duplication (bottom example) is a reversal of the order of metazoan and HGT segments on either side of the resulting chimera (58). See *SI Appendix*, Fig. S9 and *SI Text 5* and *6* for supporting evidence.

**Fig. 4.**
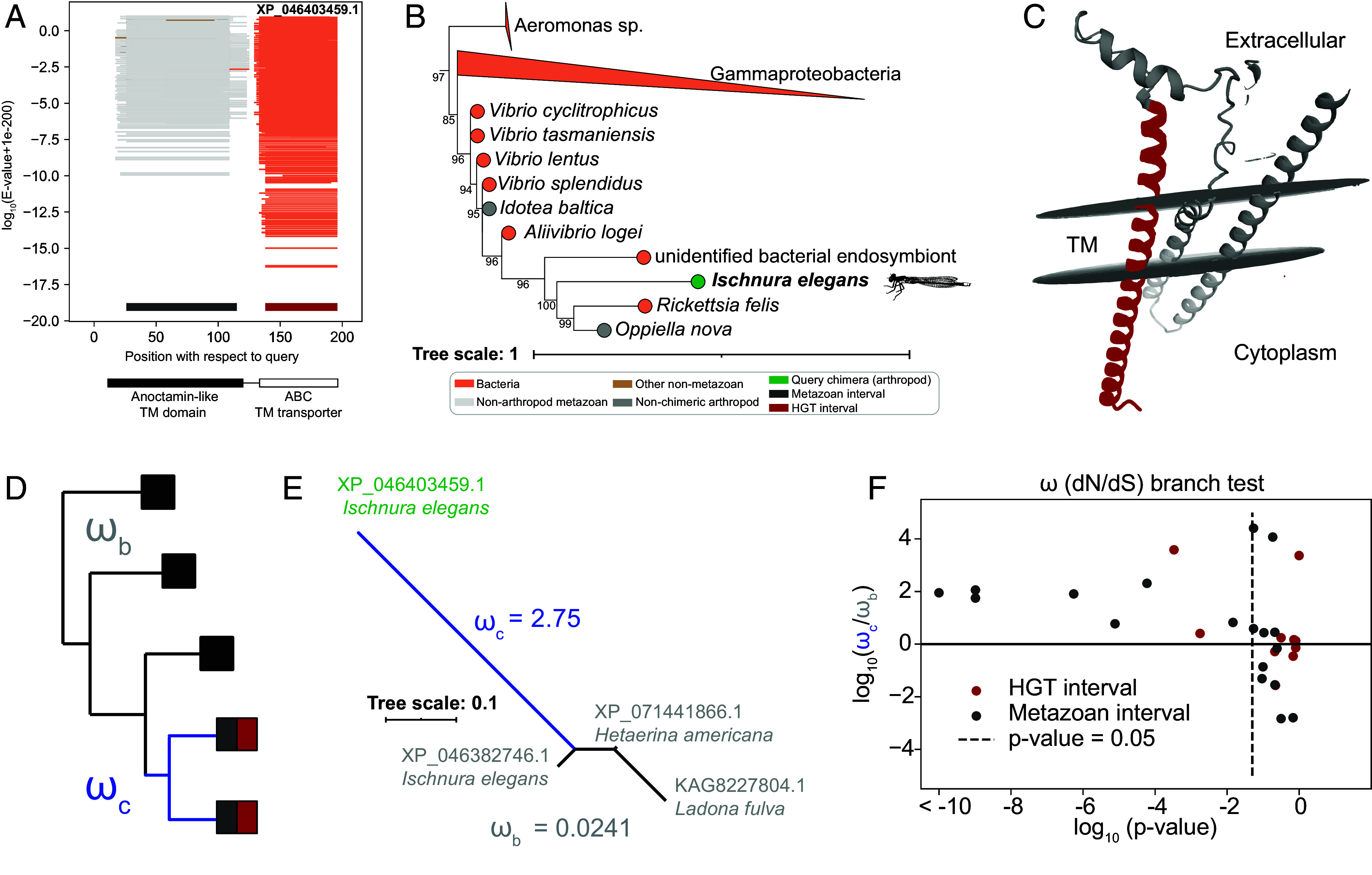
A transmembrane HGT-chimera from a damselfly illustrates postfusion neofunctionalization. (*A*) BLASTp plot excluding arthropod hits for the query chimera XP_046403459.1 from the damselfly *Ischnura elegans* (cluster 41). Metazoan ancestry is inferred for the interval (26 to 115) and HGT ancestry for the interval (138 to 196). Domain annotations are shown across the bottom of the plot. (*B*) Maximum likelihood tree for the HGT interval (138 to 196), pruned to show sequences closely related to the query chimera (full tree available on the iTOL webserver at https://itol.embl.de/shared/rkapoor) (57). Internal node values indicate ultrafast bootstrap support. Per GenBank, the “unidentified bacterial endosymbiont” was isolated from the midge *Chironomus riparius*, and **Rickettsia* felis* from the louse *Liposcelis bostrychophila*. (*C*) MembraneFold-(59) predicted transmembrane protein structure and topology for XP_046403459.1. The HGT-derived interval is colored dark red. (*D*) Schematic representation of branch test performed to compare ω values of an interval before (gray) and after (blue) an HGT-chimera-forming gene fusion event, using trees containing HGT-chimeras and their nonchimeric relatives from the same taxonomic order. (*E*) Maximum likelihood tree constructed from the metazoan interval of XP_046403459.1 and its nonchimeric phylogenetic relatives in the same taxonomic order (Odonata). ω (dN/dS) values from the branch test explained in *D* are superimposed for chimeric and nonchimeric branches. (*F*) The distribution of the fold-change in ω between chimeric and nonchimeric branches of gene trees is plotted on the y-axis, for 28 HGT-chimera intervals. Benjamini–Hochberg corrected LRT *P*-values are indicated on the x-axis.

We designed a computational pipeline to search for HGT-chimeras in arthropod genomes, which we describe in brief here and detail in *SI Appendix*, *SI Methods*. The pipeline proceeds in four broad phases (*SI Appendix*, Fig. S1). In the first phase, we assembled a search set of 7,702,369 protein inputs from 319 RefSeq scaffold or chromosome-level genomes (Dataset S1) across eight arthropod classes (*SI Appendix*, Fig. S4), excluding proteins from scaffolds <100 kb to reduce the influence of contamination. We clustered the initial search set to 610,348 proteins using MMSeqs2 to promote computational efficiency (52). In the second phase, we used the results of DIAMOND-BLASTp (53) against the NR database (54) to perform an initial screen for putative chimeric proteins. Nonarthropod BLASTp results were first used to partition query proteins into intervals of potentially distinct evolutionary history using a customized implementation of a previously described algorithm (55) (Fig. 1*A* and *SI Appendix*, Fig. S2 *A*–*C* and *SI* *File* *1*). We hypothesized that intervals with a preponderance of hits in nonmetazoan taxa and/or lower E-values for nonmetazoan than for metazoan hits were of putative HGT ancestry (right-hand interval of Fig. 1*A*), and that those whose lowest E-value hits were in other metazoan taxa or had a preponderance of metazoan hits were of ancient metazoan ancestry (left-hand interval of Fig. 1*A*). We then subjected proteins containing at least one interval of putative HGT ancestry and at least one interval of metazoan ancestry to a second round of BLASTp, using each of the two types of intervals as separate queries to further examine potential HGT and metazoan ancestry annotations from the first round of BLASTp (*SI Appendix*, Fig. S2 *D* and *E*). In the third phase, we removed potential confounders of HGT inference (ankyrin repeats and metazoan transposable elements, see *SI Appendix*, *SI Methods*) and binned HGT-chimeras into groups that we call “similarity clusters” based on their predicted protein domain architecture, using a custom approach that required all sequences in the same cluster to have the same chimeric architecture as determined by BLASTp and hmmsearch (HMMER suite) (56). Except where otherwise indicated, downstream analyses (Figs. 2–4) were performed with a single representative sequence per similarity cluster.

In the fourth and final phase of the pipeline, we assessed the support for HGT and metazoan ancestries of the distinct intervals of each cluster representative using maximum likelihood phylogenetic trees [IQ-TREE (60)] (Fig. 1*B* and *SI Appendix*, Fig. S3). To improve the sensitivity of homology detection, we constructed these trees from the results of hmmsearch (56) using profile hidden Markov models (HMMs) built from arthropod BLASTp hits as queries, or from BLASTp hits directly when <2 unique, high-confidence (*SI Appendix*, *SI Methods*) arthropod BLASTp hits were recovered (E-value for both < 1E-2). We considered proteins with at least one interval of metazoan origin, at least one interval of nonmetazoan origin, and support for tree topologies with >70% support at the relevant node (bootstrap only considered for HGT intervals, see *SI Appendix*, Fig. S3), to be candidate HGT-chimeras: genes formed by horizontal sequence transfer followed by in-frame fusion. Excluding possible within-genome paralogs (see Dataset S6 for copy numbers), we found a final set of 274 HGT-chimera genes across these query genomes, corresponding to 104 independent origination events inferred by similarity clustering (Dataset S2). Three of these 104 clusters have two predicted HGT intervals each, and one cluster has three such predicted HGT intervals. Thus, we predict a total of 109 intervals of HGT origin across these 104 clusters.

We built maximum likelihood trees for all 109 inferred HGT ancestry intervals, and observed that the trees fell into one of four topological types (Fig. 1*B*). In types I (18.3%, N = 20) and III (10.1%, N = 11), the closest relatives of the HGT-chimeras were nonmetazoan, while in types II (35.8%, N = 39) and IV (35.8%, N = 39), nonchimeric arthropod sequences were more closely related than nonmetazoan sequences. Types III and IV are also characterized by more distantly related, multisequence nonarthropod metazoan clades. We used the approximately unbiased test (61) to compare each tree in types III and IV to a constrained topology in which all metazoan sequences form a single clade (Dataset S7). This test rejected metazoan monophyly (*P* < 0.05) in favor of the HGT-supporting topology in 76.0% (38/50) of examined cases. We cannot formally exclude the possibility that a minority of inferred HGT topologies could result from phylogenetic inference artifacts or recurrent gene loss in nonarthropod lineages. However, we propose that overall, the identified HGT tree topologies are most consistent with horizontal acquisition from nonmetazoan sources.

### HGT-Chimeras Are Detected across Arthropoda.

We detected HGT-chimeras across the arthropod phylogeny (Fig. 1*D*), including members of Chelicerata and Pancrustacea. A majority (193/319, 60.5%) of examined RefSeq genomes had at least one HGT-chimera (Fig. 1*E* and Dataset S3). We note that *Folsomia candida*, the species with the greatest predicted number of HGT-chimeras, was previously reported to harbor large numbers of nonchimeric horizontally acquired genes (20, 27, 30).

To identify the broadest range of arthropod species containing each HGT-chimera (Dataset S4), we additionally searched for representatives of HGT-chimera similarity clusters in an expanded set of arthropod genomes, including an additional 197 genome annotations from GenBank (Dataset S1). We found that 81/104 similarity clusters were restricted to a single species (Fig. 1*F* and *SI Appendix*, Fig. S5*A*), a result that may reflect a relatively recent evolutionary origin or the sparse genomic sampling of many arthropod taxa (*SI Appendix*, *SI Text 1*). For similarity clusters found in more than one species, we inferred minimum evolutionary gene ages by phylostratigraphy (62) (*SI Appendix*, Fig. S5*B* and Dataset S5). The youngest HGT-chimeras (clusters 20 and 21) appeared in the congeneric microcrustaceans *Daphnia pulex* and *Daphnia pulicaria*, but were absent in the three other *Daphnia* genomes available at the time of writing. This suggests an origin for these HGT-chimeras as recently as 82,000 years ago in the *D. pulex*-*D. pulicaria* common ancestor (63). We detected a member of the similarity cluster corresponding to the gene *oskar* (cluster 1) in 181 insects spanning the subclass Pterygota, consistent with our prior finding that *oskar* originated via chimeric HGT in or prior to the last common ancestor of winged insects [>400 million years ago (MYA) (50)]. Collectively, these findings suggest that HGT-chimera formation has occurred throughout arthropod evolution.

Unexpectedly, we found two clusters with members from multiple distantly related classes of arthropods (Fig. 1*D*). Cluster 18 was found exclusively in the firefly *Photinus pyralis* (order: Coleoptera, class: Insecta) and in the mite *Oppia nitens* (order: Sarcoptiformes, class: Arachnida), a finding consistent with convergent evolution of analogous HGT-chimera architectures via independent HGT and gene fusion events (*SI Appendix*, Fig. S6). In the case of cluster 3, molecular phylogenetic analyses suggested that its sparse phylogenetic distribution could have arisen from the transfer of these HGT-chimeras between springtails, fungus gnats, and mites following the formation of this gene (*SI Appendix*, *SI Text 2* and Fig. S7). We note that intermetazoan HGT, although thought to be rare (see for example refs. 64–66) has nevertheless been reported by other studies (30, 66–68), including among these specific groups of arthropods (30, 69). Thus, HGT appears not only to have contributed to the origination of this HGT-chimera, but also to have contributed to its spread across species following its formation.

### HGT-Chimeras Are Derived from Diverse Donor Lineages.

Next, we analyzed the taxonomic identity of the sequences in the nonmetazoan clade containing each HGT interval in its respective maximum likelihood tree (Fig. 1*C* and *SI Appendix*, *SI Methods* and Dataset S7). We found that 56.9% (62/109) of HGT intervals are a part of a broader clade of bacterial sequences. Among these 62 intervals, 59.7% (37) are phylogenetically related to sequences from known arthropod bacterial symbionts, including members of the intracellular order Rickettsiales (70, 71) (genera *Wolbachia* and *Rickettsia*; Datasets S7 and S8). Further, 13 of 15 HGT intervals that nest within clades of viral sequences are phylogenetically related to sequences from arthropod-infecting viruses (Dataset S8).

We hypothesize that the relationships of HGT intervals with sequences from extant species reflect the likely phylogenetic lineage of the original donor organisms. Thus, consistent with prior analyses of insect HGT (14, 18), we propose that the majority of our HGT intervals have originated in bacteria. Although we cannot be certain of ancestral donor ecologies, the close relationships we detect with sequences from living arthropod viruses and endosymbionts could mean that symbiotic associations provided opportunities for the observed HGT events.

### HGT-Chimeras Are Transcribed.

*SI Appendix*, *SI Text 3* presents multiple bioinformatic analyses suggesting that most HGT-chimeras we predict from genome assemblies and annotations exist in their respective genomes and are expressed as functional gene products. For 41 chimeras from 20 species for which we could obtain tissue samples, we used RT-PCR and Sanger sequencing to test for the existence of continuous mRNA transcripts containing both predicted HGT and metazoan intervals in the predicted organisms (*SI Appendix*, Fig. S8 *C* and *D* and *SI File 2* and Dataset S13). We detected transcripts of 87.8% (36/41) of examined HGT-chimeras, corresponding to 24 distinct similarity clusters across 18 species. This provides strong evidence that the observed HGT-chimeric transcripts are genuine rather than artifacts of genomic contamination, bioinformatic misassembly, or misannotation.

We additionally leveraged a variety of in silico tools to predict that HGT-chimeras may have diverse functions, from nucleotide interaction to carbohydrate metabolism (Fig. 1*G*, *SI Appendix*, Fig. S8*D*, and Datasets S14 and S15). In the following sections, we use four RT-PCR-validated examples to illustrate the diverse origins, evolutionary trajectories, and predicted functions of these genes.

### A Shrimp Chimera Illustrates That Recently Evolved HGT-Chimeras Can Come under the Influence of Purifying Selection.

HGT-chimera cluster 12 (representative sequence XP_037790819.1) is found in three species of the genus of marine shrimp *Penaeus* (order Decapoda). HGT intervals of these chimeras are phylogenetically nested within a clade of bacterial sequences with high statistical support (Fig. 2*B*; tree topology tests in *SI Appendix*, Fig. S10 and Dataset S7), supporting a bacterial origin. We predict that the HGT interval is an amidase domain with intact catalytic residues (*SI Appendix*, Fig. S11), and the metazoan-derived interval is annotated as a putative growth factor receptor. This unusual domain architecture (Fig. 2*A* and *SI Appendix*, Fig. S11*A*) juxtaposes a metazoan cell signaling domain with a bacterial hydrolase in a likely soluble and extracellular protein [DeepLoc (72) probabilities > 0.76 and > 0.71, respectively; SignalP (73) likelihood > 0.99].

The three shrimp-derived sequences are monophyletic in both the HGT and metazoan interval trees (Fig. 2*B*), supporting a single origin of this chimera as recently as 83.1 MYA (*SI Appendix*, Fig. S5*B*) (74). We can further place an upper bound on this chimera’s age due to its absence in other examined decapod genomes, including *Penaeus japonicus*, which diverged from its chimera-bearing congeneric relatives 114 MYA. Despite its recent origin, this chimera has come under the influence of purifying selection, with gene-wide dN/dS = 0.29 [likelihood ratio test (LRT) *P*-value 1.06E-45] and no evidence of relaxed constraint in HGT-derived vs. non-HGT codons as assessed by fixed-sites (partition) models.

Similar patterns hold across the broader set of 23 HGT-chimeras found in more than one species: all have gene-wide dN/dS < 1, with neutrality rejected in 21 cases (Fig. 2*E* and *SI Appendix*, Table S1). Only three clusters showed significant relaxation of constraint in HGT vs. non-HGT codons after FDR correction (Fig. 2*F* and *SI Appendix*, Table S2). Consistent with our inference that 18.3% (19/104) of HGT-chimeras have been conserved for more than one million years (Dataset S5), this paucity of relaxed constraint supports the interpretation that many HGT-chimeras are functionally expressed and that horizontally acquired regions contribute to their activity.

### A Copepod Chimera Illustrates Deep Conservation and Functional Similarity among HGT and Metazoan Intervals.

We found representatives of cluster 14 (representative sequence XP_059092480.1) in the genomes of two species of microcrustaceans, *Eurytemora *carolleeae** (order Calanoida) and *Tigriopus californicus* (order Harpacticoida), implying an origin at least as ancient as the last common ancestor of extant copepods ~446 MYA (Fig. 2*D*). Further supporting our inference of deep functional conservation, we detected representatives of this cluster in transcriptomes of 6/7 additional copepod species across two orders.

The HGT interval of this chimera is phylogenetically adjacent to putative bacterial chitin deacetylase sequences (Fig. 2*D*), and shares intact catalytic residues with its *Vibrio* relative (Fig. 2*A* and *SI Appendix*, Fig. S12 *A*–*C*). The metazoan-derived region is annotated as a peritrophin (*SI Appendix*, Fig. S12*D*), a class of extracellular chitin-binding proteins that have been implicated in copepod transcriptional responses to *Vibrio* (75). Future work should examine whether this chimera also functions at the host–microbe interface (*SI Appendix*, *SI Text 4*).

The shared chitin-interacting functionality in the HGT and metazoan intervals of this chimera illustrates a broader pattern. Despite the distinct origins of their components, 36.5% (38/104) of HGT-chimeras displayed putative functional similarity in their HGT- and metazoan intervals, including eight cases in which both HGT and metazoan intervals were predicted to bind to or chemically modify carbohydrates (Dataset S15). These observations, coupled with the repeated observation of fusions of functionally related genes in other studies (4, 51, 76), could mean that HGT-chimeras formed from functionally similar components may be more likely to encode beneficial functions, contributing to their fixation and long-term preservation under purifying selection (4).

### A Mosquito Chimera Illustrates That Preexisting Nonchimeric HGT Genes May Contribute to HGT-Chimera Formation.

HGT-chimera cluster 9 (representative sequence XP_021699539.1) is found in three closely related species of mosquitoes (order Diptera), suggesting a minimum age of 92 MYA (74). It consists of a metazoan C2H2 zinc finger region and three tandem HGT-intervals, all annotated by CENSOR (77) as fragments of the auxiliary (nontransposase) protein of fungal Harbinger transposons (Fig. 3*A*). Tree topologies confirm a close relationship of the latter with fungal sequences along with mixed clades of fungal, plant, and oomycete sequences, consistent with documented cross-taxon transposon mobility (41, 78, 79) (Fig. 3*B*). Notably, the mosquito chimeras form a single clade within a broader group of nonchimeric sequences from diverse dipteran species that shared a common ancestor 241 MYA (74). These observations suggest that the HGT sequence was initially nonchimeric after acquisition by a dipteran ancestor, followed by a more recent fusion event in a mosquito ancestor (Fig. 3*C*). Across the broader set of HGT-chimeras, we find analogous tree topologies consistent with the hypothesis that nonchimeric HGT genes precede HGT-chimera formation in 38.5% (42/109) of HGT intervals (*SI Appendix*, *SI Text 5* and Dataset S10).

Both auxiliary Harbinger proteins (80) and C2H2 zinc fingers (81) are DNA binding, and we predict intact DNA-binding residues in both the HGT- and metazoan-derived intervals of this HGT-chimera (*SI Appendix*, Fig. S13), along with an intact nuclear localization sequence (DeepLoc nuclear probability > 0.90). Coupled with gene-wide purifying selection (dN/dS = 0.27, *P*-value = 2.54E-90), these findings suggest that HGT-derived transposon fragments may have been co-opted into a transcriptional regulatory role, as has been found for other cases of transcription factor—transposon fusions (82, 83). We document 22 HGT-chimeras in which both HGT and metazoan intervals are predicted to function in nucleic acid-associated processes (Dataset S15), further illustrating the recurrent pattern of functional similarity raised by the chitin-interacting copepod chimera above.

### A Damselfly Chimera Illustrates the Potential Contribution of Metazoan Gene Duplication and Neofunctionalization to HGT-Chimera Evolution.

Cluster 41 (XP_046403459.1) is restricted to a single species, the damselfly *Ischnura elegans* (order Odonata). It encodes a predicted transmembrane protein (DeepLoc probability > 0.99), containing a fragment of a metazoan anoctamin transmembrane transporter fused to a fragment of an ABC transporter likely derived from bacterial endosymbionts (Fig. 4 *A* and *B*). Using MembraneFold (59), we predicted two transmembrane helices of metazoan origin followed by a C-terminal HGT-derived transmembrane helix (Fig. 4*C*). This chimera therefore illustrates the assembly of a novel transmembrane protein via fusion of parts of transmembrane proteins from different domains of life.

The metazoan interval tree further shows that the closest relative of this chimera is a nonchimeric anoctamin gene in the *I. elegans* genome (Fig. 4*E*). Thus, we posit that duplication of a metazoan anoctamin gene facilitated the fusion of one duplicate with an HGT gene (Fig. 3*C*). We observed a significant elongation of the branch of this tree leading to the HGT-chimera relative to the remaining damselfly branches, suggesting that the fusion event was followed by accelerated sequence evolution. Consistently, we fit a two-ratio dN/dS model (84–86) to this metazoan interval tree and found that the nonchimeric metazoan sequences have evolved under strong purifying selection (dN/dS = 0.0241), while the HGT-chimera diverged under the influence of strong positive selection (dN/dS = 2.75, LRT *P*-value = 1.1E-10).

These molecular evolutionary features are shared with multiple HGT-chimeras. We find tree topologies in which either HGT or metazoan chimera intervals have close nonchimeric relatives from different genes in the same genome for 53.8% (56/104) of HGT-chimeras (*SI Appendix*, Fig. S9 *F*–*H*). Thus, gene duplication of HGT and/or metazoan genes may frequently contribute to HGT-chimera formation (*SI Appendix*, *SI Text 6*). Across species- or genus-specific HGT-chimera intervals with nonchimeric phylogenetic relatives in the same genome and in other genomes from the same taxonomic order, 64.3% (18/28) have higher dN/dS on the chimeric branches. A higher dN/dS on chimeric vs. nonchimeric branches is statistically supported by an LRT in 32.1% (9/28) of such intervals (Fig. 4 *D*–*F* and *SI Appendix*, Table S3), a result not attributable to dS saturation (*SI Appendix*, *SI Text 7*). This suggests that accelerated protein sequence evolution may be a common fate of incipient HGT-chimeras, consistent with the functional divergence (neofunctionalization) observed in other novel genes that have evolved through gene fusion and/or duplication (4, 7, 8, 31, 34, 87).

Postfusion functional divergence has been experimentally shown for the HGT-derived domain of the HGT-chimeric gene *oskar*. The closest bacterial relatives of *oskar*’s HGT-derived domain, called the OSK domain (48), are lipid hydrolases (44, 49, 50), but in insects this domain lacks key hydrolytic residues and instead binds mRNAs (48, 88). Thus, HGT-chimeras reflect evolutionary innovation not only by the juxtaposition of preexisting sequences from different domains of life, but also through postfusion sequence divergence.

## Conclusions

Novel protein structure and function can emerge from several elementary events, including amino acid substitutions, gene duplication, gene fusion, and HGT (3). In this study, we have shown that multiple of these elementary events can operate together in the origins of a previously understudied class of genes that we call HGT-chimeras. In at least 104 independent events across the history of the arthropods, HGT-chimeras evolved via the fusion of endogenous genes of ancient metazoan ancestry with genes acquired via horizontal transfer from nonmetazoan sources. Sequence duplication within the host genome played a plausible role in the formation of many HGT-chimeras (Figs. 3*C* and 4*D* and *SI Appendix*, Fig. S9), and we additionally detected a signature of accelerated amino acid substitution following HGT-chimera birth (Fig. 4*D*). Collectively, these findings reveal a rich posttransfer history of horizontally acquired sequences in arthropods.

We propose that although chimeric genes of this nature have been reported rarely (21, 39–42, 50, 51) despite significant prior work on HGT (12–16, 18, 19, 22, 24–29), we were able to uncover these HGT-chimeras by better demarcating intervals of distinct history (Fig. 1*A* and *SI Appendix*, Fig. S2). HGT-chimeras are unlikely to be detected by traditional HGT detection methods, which assume a common evolutionary history throughout the length of the open reading frame. In almost half (41/104) of HGT-chimeras, a naive interpretation of gene-wide BLASTp results via the same criteria used to infer interval ancestry would lead to a false inference of gene-wide metazoan ancestry (*SI Appendix*, Table S4). For the remaining genes, constructing a single gene tree for genic intervals with distinct histories would be methodologically problematic and, even if it resulted in a sensible topology, would obscure the true chimeric history of the gene. We expect that the application of methods like the one we describe here, that allow for intragenic phylogenetic discordance, could lead to the discovery of more HGT-chimeras, especially in lineages with frequent HGT such as grasses and parasitic plants (89, 90), fungi (91, 92), and prokaryotes (3, 12).

The biological significance of HGT has been questioned by some researchers, as many transferred segments show evidence of pseudogenization (15, 22, 93), and significant barriers to the transfer and functional integration of genes from divergent lineages have been posited (11, 16, 94–96). Motivated by the functionally coherent fusions discussed in the examples above (Figs. 2–4), we speculate that fusion with preexisting gene sequences might facilitate the integration of novel horizontally transferred sequences into preexisting functional networks. Many HGT-chimeras show signatures consistent with biological function, including multiple lines of evidence for active transcription (*SI Appendix*, Fig. S8 and Datasets S12 and S13) and gene-wide purifying selection (Fig. 2 *E* and *F* and *SI Appendix*, Tables S1 and S2). Experimental studies in diverse model systems have demonstrated important functions for many young and species-specific genes (6, 10, 33, 97–99). Thus, we consider it plausible that even the young HGT-chimeras detected here may play important biological roles. Our in silico functional predictions (Datasets S14 and S15) provide insights into the potential range of biological processes impacted by HGT-chimeras, warranting experimental validation in future studies.

To conclude, our computational approach has revealed that fusion of horizontally acquired sequences with endogenous ones has been widespread throughout the history of arthropods, illustrating a previously underappreciated versatility in evolutionary innovation via genomic “bricolage” (100).

## Methods

Software used in the “Development of an HGT-chimera detection pipeline” section included HMMER v3.3.2 (56), MUSCLE v5.1 (101), trimAl (102), and iTOL v5 (103). dN/dS ratios were computed with PAL2NAL v14.1 (104) and PAML v4.10.0 (86), with LRT details provided in *SI Appendix*, Table S8. All BLASTp searches were performed with DIAMOND v2.0.15 (53). RT-PCR and Sanger sequencing were performed on arthropod samples from donations or lab cultures (Dataset S13). Full methods are provided in *SI Appendix*. ChatGPT (v.4-5.3) was used to assist with scripting from human-generated pseudocode, as well as code review and commenting. All ChatGPT-assisted code was reviewed and revised by R.R.K.

## Supplementary Material

Appendix 01 (PDF)

Dataset S01 (XLSX)

Dataset S02 (XLSX)

Dataset S03 (XLSX)

Dataset S04 (XLSX)

Dataset S05 (XLSX)

Dataset S06 (XLSX)

Dataset S07 (XLSX)

Dataset S08 (XLSX)

Dataset S09 (XLSX)

Dataset S10 (XLSX)

Dataset S11 (XLSX)

Dataset S12 (XLSX)

Dataset S13 (XLSX)

Dataset S14 (XLSX)

Dataset S15 (XLSX)

## Data Availability

Maximum likelihood trees for HGT and metazoan intervals of the final set of HGT-chimera similarity clusters are freely accessible in the public project “Arthropod HGT-chimera interval trees 8/27/2025” on the iTOL webserver at https://itol.embl.de/shared/rkapoor (57). Associated BLAST results, hmmsearch results, multiple sequence alignments, and Newick trees have been deposited at the Dryad digital repository for this study https://doi.org/10.5061/dryad.t1g1jwtdz (105). Scripts used to implement the HGT-chimera detection pipeline and downstream analysis are available on the GitHub repository https://github.com/rishabhrajkapoor/Arthropod-HGT-chimeras-2025 with commit ID 85661ae (106). Sanger-verified cDNA sequences have been deposited at GenBank, with accession numbers available in Dataset S13. All other data are included in the manuscript and/or supporting information.
